# COVID-19 Outbreak Associated with Air Conditioning in Restaurant, Guangzhou, China, 2020

**DOI:** 10.3201/eid2611.202948

**Published:** 2020-11

**Authors:** Ana M. Rule

**Affiliations:** Johns Hopkins Bloomberg School of Public Health, Baltimore, Maryland, USA

**Keywords:** 2019 novel coronavirus disease, coronavirus disease, COVID-19, severe acute respiratory syndrome coronavirus 2, SARS-CoV-2, viruses, respiratory infections, zoonoses, family cluster, outbreak, restaurant, droplet transmission

**To the Editor:** In the research letter by J. Lu et al. (*1*), the authors claim that “The air outlet and the return air inlet for the central air conditioner were located above table C (Figure, panel B).” This sentence does not describe the actual layout depicted in the Figure, in which the air conditioner is located by table C and the exhaust fan is between tables B and D.

Furthermore, the authors do not provide evidence of why “Virus transmission in this outbreak cannot be explained by droplet transmission alone.” Their discussion does not mention the possibility that persons move around and may have been infected by touching surfaces, going to the restroom at the same time, or engaging in other close contact.

It is hard to understand how the authors conclude that “… strong airflow from the air conditioner could have propagated droplets from table C to table A, then to table B, and then back to table C.” According to the figure, air flows from table C to the exhaust fan (tables B–D). The authors do not provide evidence that the exhaust fan was not working; they ignored its presence. A simple measurement of air flow would answer this question.

The fact that “… none of the staff or other diners in restaurant X were infected” is another indication that the air conditioner was probably working. Also puzzling is the authors’ conclusion that “… the smear samples from the air conditioner were all nucleotide negative. This finding is less consistent with aerosol transmission.”

The authors’ conclusion that “… in this outbreak, droplet transmission was prompted by air-conditioned ventilation” is not supported by the data provided. They further conclude that “The key factor for infection was the direction of the airflow” but do not follow the airflow to the exhaust fan.
